# Using Google Trends Data to Study Public Interest in Breast Cancer Screening in Brazil: Why Not a Pink February?

**DOI:** 10.2196/publichealth.7015

**Published:** 2017-04-06

**Authors:** Paulo Roberto Vasconcellos-Silva, Dárlinton Barbosa Feres Carvalho, Valéria Trajano, Lucia Rodriguez de La Rocque, Anunciata Cristina Marins Braz Sawada, Leidjaira Lopes Juvanhol

**Affiliations:** ^1^ Laboratory of Innovation in Therapies, Teaching and Bioproducts /LITEB Oswaldo Cruz Institute/IOC Oswaldo Cruz Foundation Rio de Janeiro Brazil; ^2^ Research Coordination National Cancer Institute Rio de Janeiro Brazil; ^3^ Department of Computer Science Federal University of São João del-Rei São João del-Rei Brazil; ^4^ Institute of Letters. Sector of English Literature Department of Germanic Languages State University of Rio de Janeiro/UERJ Rio de Janeiro Brazil; ^5^ National School of Public Health Oswaldo Cruz Foundation Rio de Janeiro Brazil

**Keywords:** Internet, cancer information seeking, breast cancer, mass screening, health communication, early detection of cancer, infoveillance, infodemiology

## Abstract

**Background:**

One of the major challenges of the Brazilian Ministry of Health is to foster interest in breast cancer screening (BCS), especially among women at high risk. Strategies have been developed to promote the early identification of breast cancer mainly by Pink October campaigns. The massive number of queries conducted through Google creates traffic data that can be analyzed to show unrevealed interest cycles and their seasonalities.

**Objectives:**

Using Google Trends, we studied cycles of public interest in queries toward *mammography* and *breast cancer* along the last 5 years. We hypothesize that these data may be correlated with collective interest cycles leveraged by national BCS campaigns such as Pink October.

**Methods:**

Google Trends was employed to normalize traffic data on a scale from 0 (<1% of the peak volume) to 100 (peak of traffic) presented as weekly relative search volume (RSV) concerning mammography and breast cancer as search terms. A time series covered the last 261 weeks (November 2011 to October 2016), and RSV of both terms were compared with their respective annual means. Polynomial trendlines (second order) were employed to estimate overall trends.

**Results:**

We found an upward trend for both terms over the 5 years, with almost parallel trendlines. Remarkable peaks were found along Pink October months— mammography and breast cancer searches were leveraged up reaching, respectively, 119.1% (2016) and 196.8% (2015) above annual means. Short downward RSVs along December-January months were also noteworthy along all the studied period. These trends traced an N-shaped pattern with higher peaks in Pink October months and sharp falls along subsequent December and January.

**Conclusions:**

Considering these findings, it would be reasonable to bring Pink October to the beginning of each year, thereby extending the beneficial effect of the campaigns. It would be more appropriate to start screening campaigns at the beginning of the year, when new resolutions are taken and new projects are added to everyday routines. Our work raises attention to the study of traffic data to encourage health campaign analysts to undertake better analysis based on marketing practices.

## Introduction

The increasing number of searches for health-related issues generates “big data,” providing meaningful research in infodemiology, which is the study of patterns and determinants of information on the Web with the purpose to inform public health and public policy [1,2]. This new concept is grounded in two approaches: supply-based infodemiology (studies the dynamics, quantity, and quality of information available on websites, media reports, blogs, tweets, etc) and demand-based (health information seeking, eg, what people are searching for on the Internet) [1]. Information-seeking behavior (measured, eg, by the frequency with which the public enters specified search terms) has been used successfully to show unexpected interest cycles and regional seasonalities. Numerous conditions and situations have been studied, from influenza epidemics [3] to seasonality of headache [4]. Previous studies suggest that infoveillance can measure the success of a campaign in driving information-seeking behaviors [5], showing significant relationships between health campaigns and information-seeking behaviors [6]. Web data surveillance holds a strong potential to lead to overlooked phenomena [2,7,8] and might increase knowledge on campaign strategies centered on timing of interest cycles. This paper works under the demand-based approach, examining patterns of public seeking for information on breast cancer early identification, like similar works that have become increasingly frequent in recent literature [6,8-10].

Breast cancer is the most common form of cancer among women all over the world, whether in high-income or in poor countries [11], accounting for 22% of the 4.7 million new cases occurring annually among females worldwide [12,13]. There is plenty of evidence that early diagnosis initiatives of breast cancer save far more lives and are much more cost-effective than treatment of late stages [14,15]. From the perception of countries like Brazil, the efficacy and adherence to breast cancer screening (BCS) is still a problematic issue from a public health policy perspective [16]. Brazilian mortality rates are increasing with striking variations between geographic regions, and several factors may account for the disparities, including delays in diagnosis due to low education level [17], low adherence to screening programs, and gaps in their implementation [18,19]. Surveillance systems databases have been used to assess self-reported cancer screening utilization. Although invaluable in identifying determinants of screening use and describing trends, these database systems are too complex and costly, and remain a challenge for the largest country in Latin America. On the other hand, a massive number of queries conducted through Google create data that can be analyzed with Google Trends, a publicly available tool used to compare the volume of Web search queries in different periods [20].

The use of search volume for predicting real-world events may have less to do with their superiority over other data systems than with matters of low cost, transparency, simplicity, and reproducibility across a variety of domains. Among other free access tools available [19], Google Trends provides essential data to public health planners as weekly reports on the volume of queries related to pertinent issues. Google Trends shows oscillations whenever a particular search term is searched for, relative to the top number of searches [20]. We hypothesize that these query data may be correlated with collective interest cycles affected by campaigns and, thus, may be suitable in “predicting the present” in terms of “BCS attitudes.” If that is the case, Google Trends would be a low-cost support for screening campaigns planners, providing feedback information almost immediately after interventions. In this paper, we studied oscillations of public interest in queries toward mammography and breast cancer along the last 5 years.

## Methods

**Figure 1:**
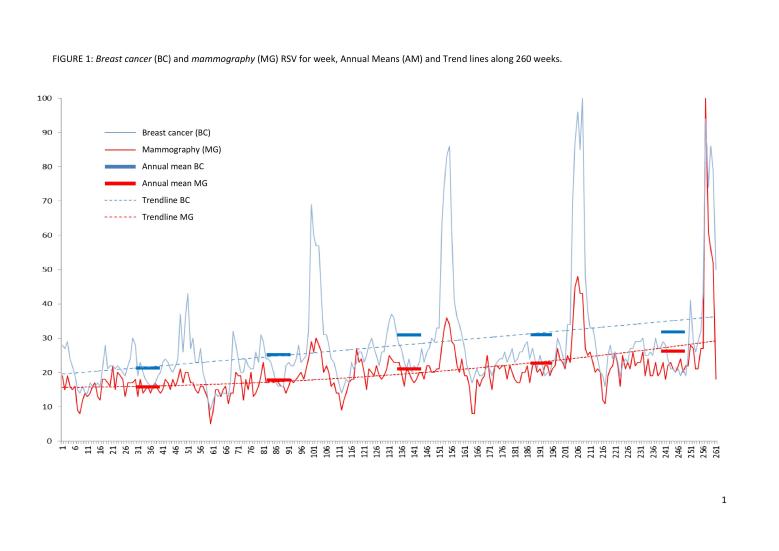
Breast cancer and mammography relative search volume for week, annual means, and trendlines along 260 weeks.

Google Trends is a Web-based free tracking system of Google search volumes. Google Trends algorithmics normalize data for the overall number of searches on a scale from 0 (search volume <1% of the peak volume) to 100 (peak of popularity), presenting them as a weekly relative search volume (RSV). RSV values are by definition, as presented in the y-axis (Figure 1), always less than 100, and display a proportion compared with the highest search volume. This approach corrects results for population size and Internet access, both of which increased during the study period.

Mammography and breast cancer (“mamografia” and “cancer de mama” in Portuguese) were used as search terms to produce separate time series (put together in Figure 1) in the last 260 weeks (November 2011 to October 2016), with the filter “Brazil” (country) in category “Health.” We selected these search terms based on their face validity, excluding their plural forms or any other unusual forms, which resulted in low weekly RSV.

The results were analyzed considering the data export through comma separated value (CSV) files. The weekly and monthly RSV values were compared with annual means, and a graph was plotted adding up annual means to highlight differences between weekly RSV series for both terms. Polynomial trendlines (second order) were added to the weekly RSV to estimate trends over the 261 weeks.

## Results

Results show an upward trend for both breast cancer and mammography searches over the 5 years, with almost parallel trend curves (Figure 1). The annual means on breast cancer queries show a slight decline between 2011 and 2012 (not so relevant, considering just the last two months, without Pink October) followed by a raise from there, with a “jump” in 2015 annual means, as shown in Table 1. Annual means of mammography searches rose steadily along the 5 years (Table 1). Interest in breast cancer seems to significantly increase in Pink October months with remarkable higher means (reaching up to 196.8% above the 2015 annual means). There were several minor peaks throughout the years (without impacts comparable with Pink October months and no obvious seasonality). Likewise, there were remarkable growing peaks for mammography searches along Pink October months (reaching 119.1% above the 2015 annual means), though not so “instable” throughout the years when compared with breast cancer. A short downward trend along December-January months was also noteworthy along the 261 weeks—mammography reached 27.1% and breast cancer 36.6% below the annual means. These oscillations traced an N-shaped curve with higher peaks in Pink October months and sharp falls along the two subsequent months (Figure 1).

**Table 1:** Breast cancer and mammography relative search volume along Pink October and December-January (means) related to annual means.

Relative search volume		2011	2012	2013	2014	2015	2016^a^
**Mammography**							
	Annual means (std)		15.8 (2.9)	17.8 (4.5)	21 (5.2)	22.7 (7.4)	26.2 (14.6)
	Pink October (annual means)		18.5 (↑17.1%)	27.5 (↑54.5%)	33 (↑57.1%)	44.8 (↑97.4%)	57.4 (↑119.1%)
	December-January means (annual means)	13 (↓11%)	12.7 (↓19.6%)	14.8 (↓16.9%)	15.3 (↓27.1%)	19.3 (↓15%)	
**Breast cancer**							
	Annual means (std)		21.3 (6.3)	25.2 (12)	30.9 (15.4)	31 (19.7)	31.8 (17.6)
	Pink October (annual means)		33 (↑54.9)	54.3 (↑115.5%)	76 (↑146%)	92 (↑196.8%)	76.6 (↑140.9%)
	December-January means (annual means)	16.3 (↓26.9%)	13.9 (↓34.7%)	18.8 (↓25.4%)	19.6 (↓36.6%)	22.8 (↓26.5%)	

^a^From January to October.

## Discussion

### Principal Findings

In this study, we examined the utility of Google Trends in identifying cycles of public interest in breast cancer and BCS. Although Internet access is still concentrated in metropolitan areas in Brazil, limiting Google Trends’ use in areas with a low search volume, several studies seem to support the assumption that queries are sensible to foresee collective movements in real life. This is a well-known truism in marketing sciences grounded on studies on Google Trends’ power in “predicting the present” [21], meaning that search volume correlates with contemporaneous events. The same rationale is being employed in several health research fields, and it has been useful to elucidate a wide range of questions from vaccination compliance [22], to protection against ultraviolet exposure during summer season [23], and interest in cancer issues after prevention campaigns [24].

There are two points to be highlighted in the results: (1) the growing interest in the early identification of a major public health problem and (2) the short collapse of this interest cycle at the end of each year (Table 1). In short, our results showed N-shaped RSV curves both in mammography and breast cancer (Figure 1), with higher peaks along Pink October months and sharp declines along December and January. This “cancer screening vortex” has been also described by Schootman and colleagues—the highest RSV along breast, colorectal, cervical, prostate, and lung cancer screening campaigns and the lowest during December-January [10]. In this case, this gap may be due to the Brazilian cultural aspects concerning summer vacations, Christmas, and New Year’s celebrations. People tend to disregard issues related to illness and death, typically postponing some health decisions for the next year.

In Brazil, Pink October’s strategy has been planned to promote collective interest in BCS in the context of cultural taboos and misconceptions. In fact, interest in breast cancer seems to significantly increase in October, although with several peaks throughout the year, with no evident seasonality. Likewise, in recent years, RSV concerning the early diagnosis of breast cancer has been significantly higher along Pink October months. It seems to be growing almost exponentially, and perhaps will go beyond searches on breast cancer in the next few years. It is consistent, with several works describing the use of Internet (boosted by higher educational level and the worldwide widespread of mobile phones) as a resource to self-care [25,26]. There is also a close correlation between the level of education—which has grown in Brazil in the last decades [27]—and accesses by Google to issues concerning science and health [28]. Interest in breast cancer always outperformed (in absolute and relative terms) mammography, but showed erratic patterns over the years and irregular growth in annual means. This may be consequent to events without seasonality, linked to the high incidence of new cases and constant media coverage—especially among celebrities who seem to boost the number of hits [29,30].

Surveillance systems databases have been very useful to assess self-reported cancer screening utilization. These data have been invaluable in identifying determinants of screening practices and describing trends and regional inequalities over time [31,32]. Unfortunately, due to the need of massive survey interviews for data collection, these database systems are too costly for low-income countries [33]. The complexity of a suitable survey structure required to aggregate reliable data, requiring the participation of a large study population, is also a huge obstacle for the largest country in Latin America. Methodological problems are also involved—public health planners must consider accuracy problems caused by self-report questionnaires and selection bias [32]. As a result, the Brazilian population–based prevalence of cancer screening methods are not precise and the cultural impact of Pink October campaigns concerning BCS behavior is still unknown. Schootman and colleagues [10] examined the utility of Google Trends relative to a surveillance system focused on cancer screening (behavioral risk factor surveillance system). Social interest in learning about cancer screening exams was compared with surveillance systems based on self-reported use of these tests. In the same manner, the present results are eloquent to point out that attention has been increasingly drawn to the means of early identification of cancer. It is not clear if these findings may be taken as a plain evidence of well-succeeded campaigns supported by huge Brazilian government investments in access to screening [34] leveraged by the raise in educational levels [27] and widespread use of Google in mobile phones, tablets, notebooks, and desktops [17]. It is not possible to be sure if women moved forward from curiosity in Google queries to effective action. Nonetheless, the number of mammograms performed in the Brazilian Public Health System has jumped to just over 2.5 million (61.9% growth) in the period studied [34].

#### Timing-Based Strategies and the “Cancer Vortex”

There are several reports in the literature concerning campaigns and health interventions based on “what” and “how” (selection of qualified information and proper vehicles to deliver messages) [35,36], “where” (environments in which campaigns would be more effective) [37,38], and “who” (who are the best counselors) [30,39,40]. Nonetheless, reports based on “when”—the ideal timing for intervention—are not so frequent. Given the described findings and considering that the effectiveness of campaigns may be influenced by their impact and persistency in everyday life (measured in terms of “intensity,” “duration,” and convergence with relevant facts), it would be reasonable to consider some changes in Pink October timing. It would be reasonable to assume that, in Brazil, the anticipation of “Pink October” to the beginning of each year could extend the beneficial effect of the campaigns. Considering that both RSV curves decline sharply in December and January of each year (consistently with other authors in other continents [10]), would it be reasonable to expand the beneficial effects of Pink October by adding some months between its interest peak and the “December-January cancer vortex”? If we go further in this perspective and change to “Pink February,” would more people be interested in BCS for a longer period of time? Following this reasoning, it would be more appropriate to have screening campaigns at the beginning of the year, when new resolutions are taken and new projects are added to everyday routines.

### Limitations

In Brazil, Web access is still concentrated in (but not limited to) metropolitan areas, which would limit the use of Google Trends in rural areas or regions with a low search volume. In fact, specific subpopulations and their cultural disparities may not be reachable by RSV algorithmics. In addition, Google Trends data only represent searches performed in Google. It is also important to consider that, although it represents a simple and low-cost alternative to nationwide screening database, Google Trends is still insufficient to describe screening behavior peculiarities at a global level. Nonetheless, as mentioned before, several works have described information-seeking behavior as a proxy of self-care attitudes. The potential of Google Trends to generate hypotheses about public awareness and interest in multiple aspects of cancer is also well documented [6,9,10].

### Future Work

Future studies based on algorithmics sensible to interest cycles among small community groups should be useful to plan interventions tailored to the local needs. Study designs and analytic tools more appropriate to estimate the effects of media coverage on screening behavior would also be of invaluable help.

### Conclusions

The leading goal of this study is to raise attention to forecasting methods using massive data to encourage health policy makers to undertake more sophisticated analyses based on classic marketing practices. Timely evaluations after campaigns may inform policy makers about awareness and interest seasonal cycles, which would leverage further interventions. Transparency of methods, simplicity, and reproducibility make the use of these new approaches an important alternative for low-income and huge-dimension countries. Timing-based strategies and Google Trends evaluations after campaigns may inform policy makers about seasonal cycles of attention and interest, which would leverage further interventions. We believe that patterns described here can be useful as baselines to help campaign analysts get started with specialized techniques that can be subsequently employed in more effective campaigns. The understanding and proper use of Google Trends oscillations, even being common sense for marketing researchers, are challenging for disciplines like public health, where government agencies work with a different concept of timing and public health demands. However, RSV trends should be clear for public communication planners with broad perspectives and committed in a timely fashion with users’ demands.

## Abbreviations

BCS: breast cancer screening
CSV: comma separated values
RSV: relative search volume
